# Incidence of Acute Myocardial Infarction in Patients Presenting With Cerebrovascular Accident in a Tertiary Care Centre in Eastern India

**DOI:** 10.7759/cureus.29005

**Published:** 2022-09-10

**Authors:** Jaymala Mishra, Abhay Kumar, Santosh Kumar, Siddharth Singh, Santosh Kumar Nayan, Anand Dev

**Affiliations:** 1 Emergency Medicine, Indira Gandhi Institute of Medical Sciences, Patna, IND

**Keywords:** acute myocardial infarction, st-elevation myocardial infarction (stemi), ck-mb, transient ischemic attacks, cerebro-vascular accident

## Abstract

Acute myocardial infarction in individuals who have had a cerebrovascular accident or transient ischemic attack (CVA-TIA) is a medical emergency, which must be examined and treated as soon as possible. Physicians face a significant problem in managing this scenario because early treatment of one ailment would surely postpone treatment of the other. Early detection and treatment will have an impact on the patient's morbidity and mortality in the future, as well as aid in the patient's rehabilitation. On the basis of ECG alterations and cardiac biomarkers, a prospective observational study was conducted in 103 diagnosed CVA patients to investigate the incidence of myocardial infarction. Infarct and hemorrhagic CVA cases were evenly distributed. According to the age-based distribution, the highest rate of myocardial infarction (8%, 8) was observed in those aged 51-60 years. The male-to-female ratio is 1.86:1. Thirty-two patients had diabetes, among them 75% had only elevated creatine kinase MB (CKMB) with no myocardial infarction (MI), whereas 59 patients had hypertension of which 70% had only elevated CK-MB with no MI. ST-elevation myocardial infarction (STEMI) with high CKMB accounted for 14.78% (15) of the cases, but the majority (71%, 73) of the cases had elevated CKMB with no MI, and the rest presented with normal CKMB. Elevated CKMB with or without STEMI serves as a poor prognosticating factor. Therefore, these patients should be managed on a priority basis for a better outcome.

## Introduction

Acute coronary syndrome and cerebrovascular accident (CVA) are both medical emergency that needs to be diagnosed and treated side by side. Acute ischemic stroke has been shown to increase the incidence of acute coronary syndrome and vice versa [1-3]. Patients with transient ischemic attack have a higher relative risk of myocardial infarction (MI) than the general population [4]. According to a study, 13.7% of individuals with acute ischemic stroke exhibited an increased cardiac biomarker [5]. In the absence of primary cardiac issues, myocardial damage was found following ischemic stroke, the pathogenesis of which is unknown. More than 50% of coronary stenosis was found in up to one-third of patients with ischemic stroke who had no previous history of cardiac symptoms. Following an acute ischemic stroke, there is a substantial chance of recurrence; nevertheless, myocardial infarction is the major cause of death in these patients [4, 6, 7]. Treating such patients is extremely difficult due to the lack of a clear guideline in them. Patients presenting with acute MI with concomitant CVA-TIA (cerebrovascular accidents or transient ischemic attacks) exhibited a complicated hospital course, with a mortality rate of 41%-61% [8,9]. The goal of this study was to determine the prevalence of myocardial infarction in patients who had a cerebrovascular accident, as well as the demographic profiles and risks associated with it.

## Materials and methods

The study adhered to the tenets of Helsinki and was approved by the Institutional Research Ethics Committee (248/IEC/IGIMS/2021). Informed consent was taken. Confidentiality of information was assured. This was a single-centered prospective observational study. The duration of the study was from October 2021 to March 2022. Sample size of 96 was calculated using Leslie Kish formula, n = Z^2^pq/d^2^ where p is proportion = 0.5, q is 1-p that is 0.5, d is precision taken as 10%, z is standard normal deviation which is 1.96, therefore n = 96. Hence a minimum of 96 cases needed to be enrolled in the study.

Patients presenting to the emergency department with altered sensorium and CVA were included in the study. The diagnosis of stroke/TIA was based on the onset of a localized neurological loss that lasted for at least 24 hours (stroke) or recovering earlier (TIA) [9]. A CT/MRI scan of the brain was performed for a definitive diagnosis to identify ischemia/hemorrhage as the cause of the CVA. ECG and cardiac biomarker (CK-MB) were done for every study subject. Myocardial infarction was defined on the basis of ECG findings: ST-segment elevation of at least 2 mm in two contiguous precordial leads and 1 mm in two adjacent limb leads or new left bundle branch block (LBBB) or development of pathological Q waves in the ECG was suggestive of ST-elevation myocardial infarction (STEMI) whereas for unstable angina or non-ST-segment elevation MI criteria were transient ST elevation, ST depression, or new T wave inversion. Chronic kidney disease patients with baseline elevated CK-MB enzymes were excluded from the study.

CK-MB was estimated using enzymatic immune inhibition test for the quantitative determination of the creatine kinase-MB isoenzyme in human serum and plasma on Beckman Coulter analysers and the normal reference range is 0-25 U/L. For all patients with a confirmed CVA, demographic data such as age and sex, as well as historical data such as diabetes mellitus, hypertension, and coronary artery disease, were obtained during the trial.

Categorical variables were compared across groups and expressed as a number and percentage of patients. Pearson’s chi-square test for independence of attributes/Fisher’s exact test as appropriate was performed. Continuous variables were expressed as mean ± standard deviation and compared across groups using unpaired t-test/One Way ANOVA test if the data followed a normal distribution and Mann-Whitney U test/Kruskal Wallis test if the data did not follow a normal distribution. The statistical software SPSS version 20 (IBM Corp., Armonk, NY) was used for the analysis. An alpha level of 5% was taken. A P-value less than 0.05 was considered as significant.

## Results

The study included 103 patients who had suffered a cerebro-vascular event. According to their CK-MB values, patients were divided into three subgroups: normal (15), elevated with no MI (73) and ST-elevation myocardial infarction (STEMI) with elevated CK-MB (15) (Table 1).

**Table 1:** Types of CVA in relation with CKMB and myocardial infarction CVA - Cerebrovascular accident; CKMB - Creatine kinase MB; MI - Myocardial infarction; STEMI - ST-segment elevation myocardial infarction.

Types of CVA	Normal CKMB	Elevated CKMB without MI	STEMI with elevated CKMB
Infarct	7	36	8
Hemorrhagic	8	37	6
Infarct and Hemorrhagic	0	0	1

**Figure 1:**
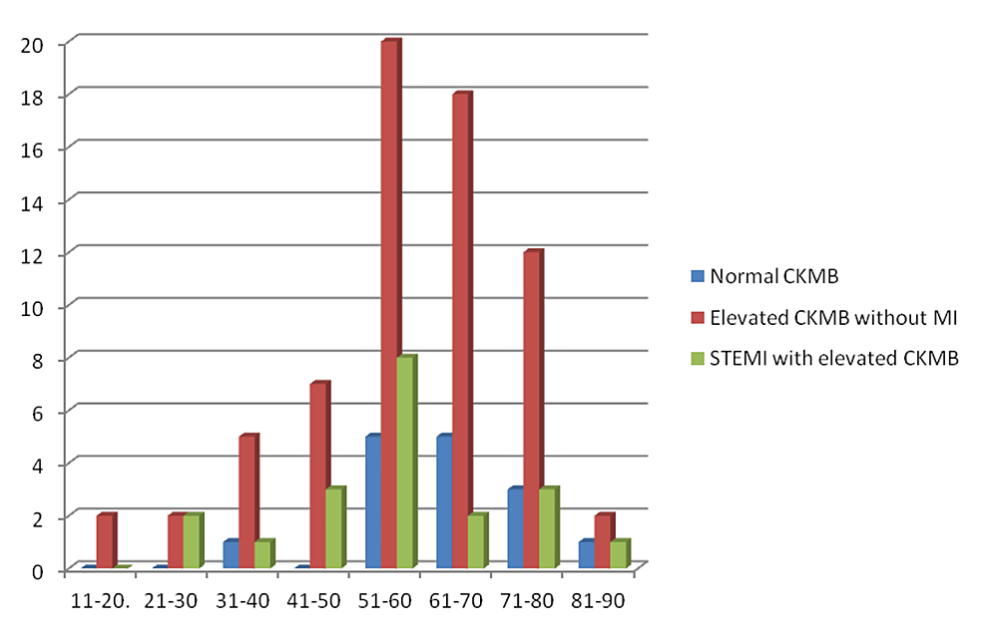
Incidence of myocardial infarction in different age groups CKMB - Creatine kinase MB; MI - Myocardial infarction; STEMI - ST-segment elevation myocardial infarction.

The age of the patients ranged from 11 to 90 years, with a mean of 59.84±14.65 years. According to the age-based distribution (Figure 1), the highest rate of myocardial infarction (8%, 8) was observed in those aged 51-60 years, followed by 2.91% (3) in those aged 41-50 years and 71-80 years, and 1.94% (2) in those aged 21-30 years and 61-70 years.

**Figure 2:**
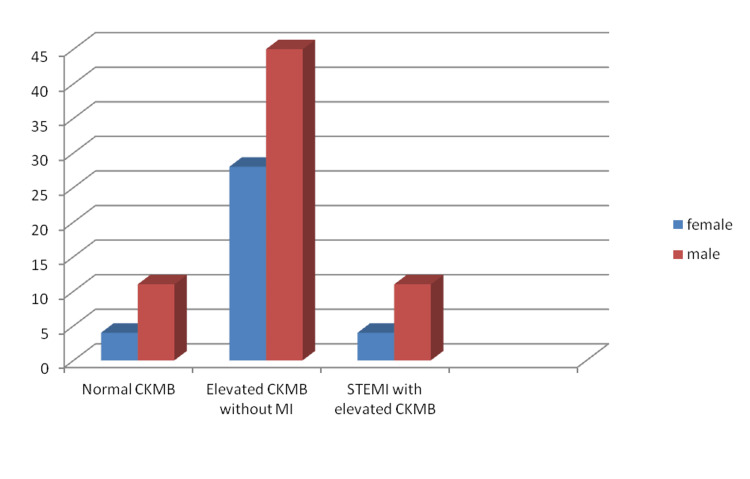
Incidence of myocardial infarction among different sex CKMB - Creatine kinase MB; STEMI - ST-segment elevation myocardial infarction; MI - Myocardial infarction.

**Figure 3:**
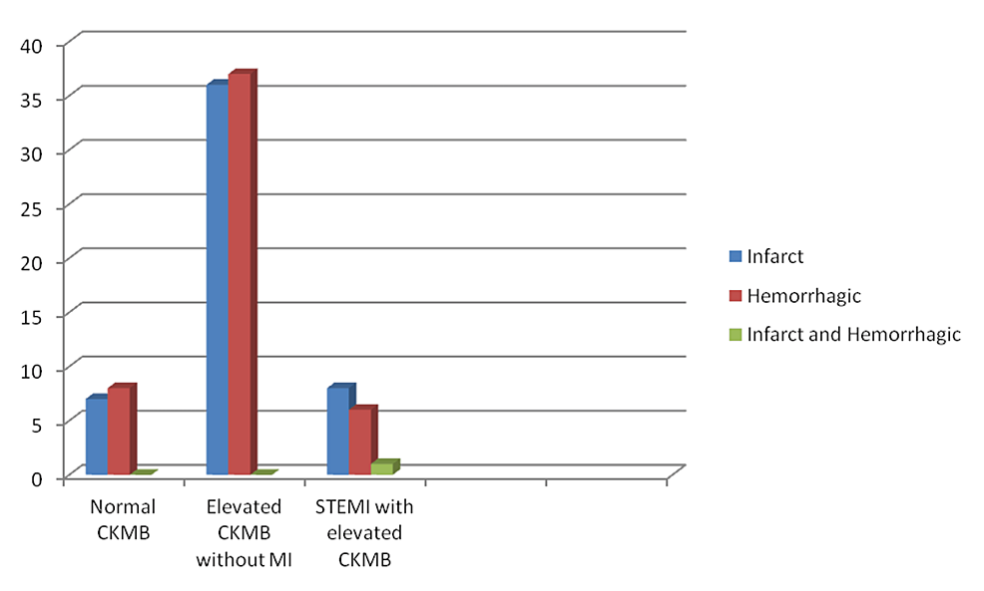
Incidence of myocardial infarction in different types of CVA CVA - Cerebrovascular accident; CKMB - Creatine kinase MB; MI - Myocardial infarction; STEMI - ST-segment elevation myocardial infarction.

There were 67 men and 36 women among the 103 patients. Gender (male/female) distribution among subgroups was as follows: STEMI with high CK-MB was 4/11, high CK-MB without myocardial infarction was 45/28, and normal CK-MB was 11/4 (Figure 2). However, the association was not found to be statistically significant (p > 0.05). Seventy percent (36) of the total 50 infarct patients had only elevated CK-MB without myocardial infarction, while 16% (8) had STEMI with increased CK-MB, and 14% (7) had normal CK-MB (p > 0.05) (Figure 3). Only 11 of the patients had a history of coronary artery disease. Seven of them had only elevated CKMB at the time of presentation and two had normal CK-MB and STEMI each.

**Figure 4:**
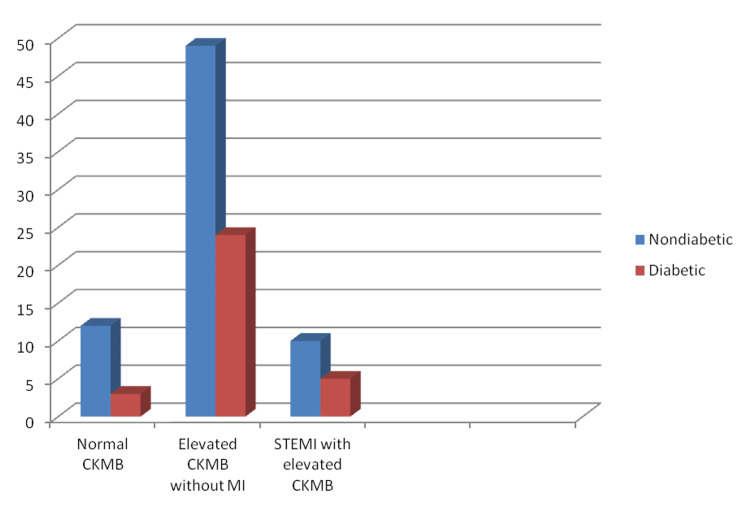
Incidence of myocardial infarction among diabetes mellitus patients CKMB - Creatine kinase MB; MI - Myocardial infarction; STEMI - ST-segment elevation myocardial infarction.

**Figure 5:**
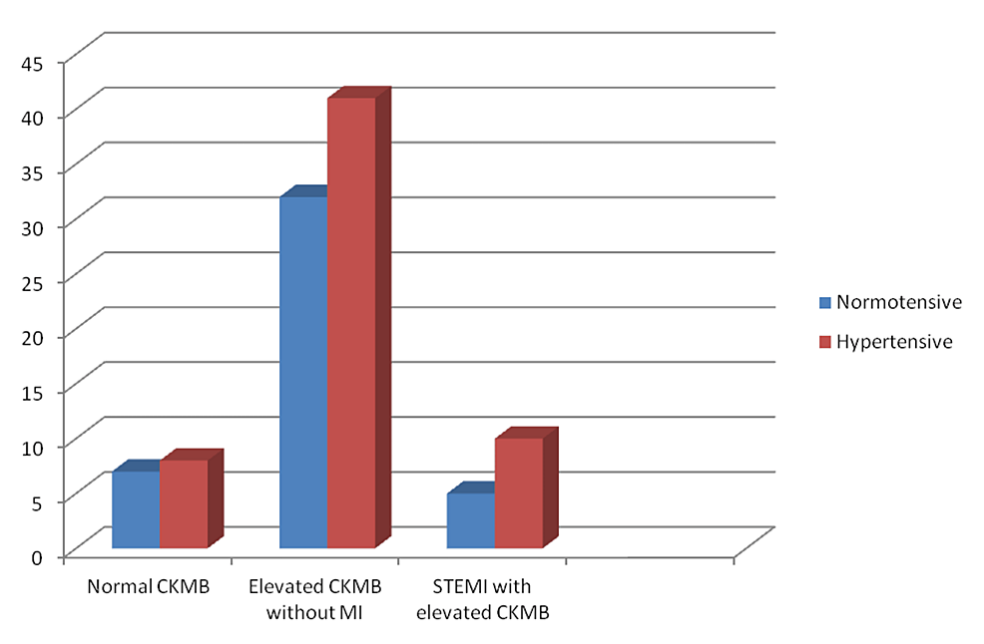
Incidence of myocardial infarction among hypertensive patients CKMB - Creatine kinase MB; MI - Myocardial infarction; STEMI - ST-segment elevation myocardial infarction.

**Figure 6:**
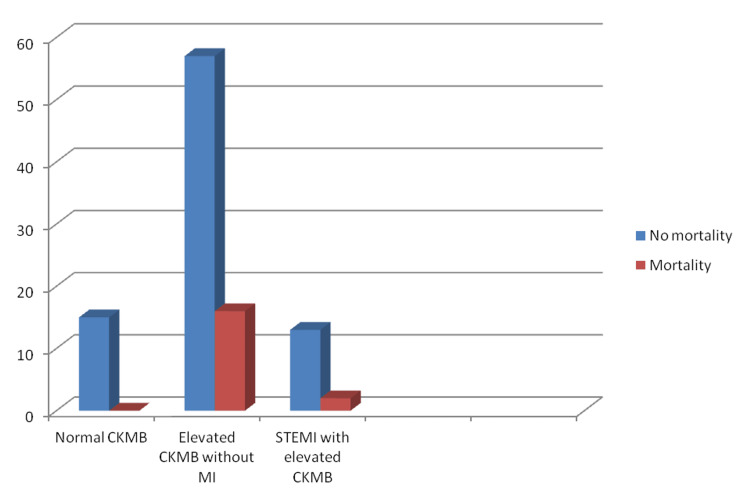
Mortality among patients with cerebrovascular accident CKMB - Creatine kinase MB; MI - Myocardial infarction; STEMI - ST-segment elevation myocardial infarction.

A total of 51 patients had hemorrhagic CVA of which 72% (37) patients had merely elevated CK-MB, 12% (6) had STEMI with elevated CKMB, and 16% (8) had normal CKMB (p > 0.05). There was one case of combined infarct and hemorrhage. A total of 32 patients had diabetes, among them 75% (24) had only elevated CKMB with no MI, 16% (5) had STEMI, and 9% (3) had normal CK-MB (Figure 4). A total of 59 patients had hypertension, among them 70% (41) had only elevated CK-MB with no MI, 17% (10) had STEMI, and 13% (8) had normal CK-MB (p > 0.05) (Figure 5). At the end of the study, total mortality was 18% (18): 16% (16) in the group with raised CKMB without MI and 2% (2) in patients with STEMI (p > 0.05) (Figure 6).

## Discussion

The purpose of this study was to determine the rate of acute myocardial infarction in patients who presented with altered sensorium due to cerebrovascular accidents in a tertiary care hospital in eastern India. Several studies have found an association between myocardial infarction and CVA. As both are emergency medical situations, they must be handled together and without delay in view of increased morbidity and mortality. Atherosclerosis is the core pathophysiology in stroke, coronary artery disease, and myocardial infarction. Any atherosclerotic event, whether in the same or a different arterial bed, increases the chance of another [10].

According to this study, the total incidence of myocardial infarction is 15%, with a maximum incidence of eight cases in the 51-60 year age group, which is consistent with the study, which found 12.7% incidence of acute myocardial infarction in cerebrovascular accident patients [11]. Gunnoo et al. found that more than half of individuals with CVA exhibit signs of asymptomatic coronary plaque and one-third have severe stenosis, defined as >50% blockage. As a result, many people without a history of ischemic heart disease (IHD) may be at risk for myocardial infarction. According to the MASS (Multiple Atherosclerosis Site in Stroke) study, 51% of coronary plaque was demonstrated in patients of stroke with no atherosclerotic plaque in cerebral arteries [2,12].

The male-to-female ratio is 1.86:1, which is similar to other studies [13, 14]. Hormonal effects (estrogen) on cerebral circulation, optimal blood pressure, and genetic factors contribute to a lower incidence of acute cerebrovascular accidents among the female population [13, 14].

In our study, infarct and hemorrhagic CVA cases were evenly distributed. The majority in both groups have increased CK-MB without any heart ailments. In patients without MI, CK-MB was found to be elevated in 24% of people in 51-60 years followed by 17% of people in 61-70 years who had CVA. Isolated elevations of CKMB were found more in CVA patients with both diabetes and hypertension. These findings were similar to a study by Mochmann et al., who reported that the majority of acute ischemic stroke patients with elevated cardiac biomarkers do not have concomitant acute coronary syndrome, and that nearly half of the patients had no signs of obstructive CAD. Also while there was angiographic evidence of a coronary culprit lesion and high troponin in around one-fourth of all patients with acute ischemic stroke, there was no underlying culprit lesion in the majority of acute ischemic stroke patients [15].

The increased levels of cardiac biomarkers in patients with acute stroke could be explained by cardiac myocyte rupture caused by sympathetic nervous system activation and injury to the insular cortex, which causes a rise in catecholamine such as adrenaline and noradrenaline. Due to disruption in capillary perfusion induced by increased platelet aggregation, high catecholamine levels in the myocardium promote calcium influx in cardiac myocytes, which compromises cardiac contractility and function. The higher level of CK-MB in brain injury is also explained by the B subunit of CK-BB. Increased CPK-MB without changes in troponin I could be the result of neurogenic effects [16-18].

There was 18% mortality in cases presenting with high CK-MB (>35 ng/ml) although the association was not found to be significant. James et al. have also reported 17% mortality in individuals with acute ischemic stroke and elevated cardiac biomarker. The increased mortality could be attributed to severe stroke in these patients. Raised Troponin T was found to have a non-significant trend with the severity of ischemic stroke. Diabetes, peripheral vascular disease, and cardiac arrhythmia are all risk factors for early MI following a stroke [19].

Routine cardiac biomarker measurement in patients with CVA is not routinely done. In this study, increased cardiac biomarkers had a non-significant trend with a poor prognosis for short-term survival and hence can be used as a prognosticating factor. As per a study done by Etgen et al., the American Stroke Association, the Scottish Intercollegiate Guidelines Network, and the National Institute of Clinical Excellence-based UK acute stroke guidelines do not recommend routine measurement of biomarkers, but it may be helpful for patients who require early intervention for secondary prevention of coronary artery disease [20-23].

The limitation of the study is the small sample size, so a larger cohort is needed to ascertain the association of mortality and cardiac biomarkers in patients with CVA.

## Conclusions

Myocardial infarction is associated with poor short-term prognosis in CVA patients, including increased mortality. Raised CK-MB with or without ST elevated myocardial infarction can serve as a marker for poor prognosis. To determine whether enhanced care, prolonged cardiac monitoring, or particular therapeutic modalities could improve the prognosis in these high-risk individuals, more research is required.

## Appendices

Following tables (Tables 2-8) have been provided as supplemental information for a better understanding of statistics.

**Table 2:** Incidence of myocardial infarction in different age groups CKMB - Creatine kinase MB; MI - Myocardial Infarction; STEMI - ST-segment elevation myocardial infarction.

Age (in years)	Normal CKMB	Elevated CKMB without MI	STEMI with increased CKMB	Total	p-value
11-20	0	2	0	2	0.619
21-30	0	2	2	4	
31-40	1	5	1	7	
41-50	0	7	3	10	
51-60	5	20	8	33	
61-70	5	18	2	25	
71-80	3	12	3	18	
81-90	1	2	1	4	
	15	73	15	103	

**Table 3:** Incidence of myocardial infarction among different sex CKMB - Creatine kinase MB; MI - Myocardial infarction; STEMI - ST-segment elevation myocardial infarction.

Sex	Normal CKMB	Elevated CKMB without MI	STEMI with increased CKMB	Total	p-value
Female	4	28	4	36	0.528
Male	11	45	11	67	
Total	15	73	15	103	

**Table 4:** Incidence of myocardial infarction in different types of CVA CKMB - Creatine kinase MB; MI - Myocardial infarction; STEMI - ST-segment elevation myocardial infarction.

Infarct/Hemorrhage	Normal CKMB	Elevated CKMB without MI	STEMI with increased CKMD	Total	p-value
Infarct	7(46.67)	36(49.31)	8(53.33)	51(49.51)	0.533
Hemorrhage	8(53.33)	37(50.68)	6(40)	51(49.51)	
Infarct with hemorrhage	0(0)	0(0)	1(6.67)	1(0.97)	
	15(100)	73(100)	15(100)	103(100)	

**Table 5:** Incidence of myocardial infarction among diabetes mellitus patients CKMB - Creatine kinase MB; MI - Myocardial infarction; STEMI - ST-segment elevation myocardial infarction; DM - Diabetes mellitus.

DM	Normal CKMB	Elevated CKMB without MI	STEMI with increased CKMB	Total	p-value
No	12(80)	49(67.12)	10(66.67)	71(68.93)	0.64
Yes	3(20)	24(32.88)	5(33.33)	32(31.07)	
	15(100)	73(100)	15(100)	103(100)	

**Table 6:** Incidence of myocardial infarction among hypertensive patients CKMB - Creatine kinase MB; MI - Myocardial infarction; STEMI - ST-segment elevation myocardial infarction; HTN - Hypertension.

HTN	Normal CKMB	Elevated CKMB without MI	STEMI with increased CKMB	Total	p-value
No	7(46.67)	32(43.84)	5(33.33)	44(42.72)	0.714
Yes	8(53.33)	41(56.16)	10(66.67)	59(57.28)	
	15(100)	73(100)	15(100)	103(100)	

**Table 7:** Mortality among patients with cerebrovascular accident CKMB - Creatine kinase MB; MI - Myocardial infarction; STEMI - ST-segment elevation myocardial infarction.

Death	Normal CKMB	Elevated CKMB without MI	STEMI with increased CKMB	Total	p-value
No	15(100)	57(78.08)	13(86.67)	85(82.52)	0.121
Yes	0(0)	16(21.92)	2(13.33)	189(17.48)	

**Table 8:** Incidence of myocardial infarction among diagnosed coronary artery disease (CAD) patients CKMB - Creatine kinase MB; MI - Myocardial infarction; STEMI - ST-segment elevation myocardial infarction; CAD - Coronary artery disease.

CAD	Normal CKMB	Elevated CKMB without MI	STEMI with increased CKMB	Total	p-value
No	13(86.67)	66(90.41)	13(86.67)	92(89.32)	0.678
Yes	2(13.33)	7(9.59)	2(13.33)	11(10.68)	
	15(100)	73(100)	15(100)	103(100)	
