# Correction: Incidence of Acute Myocardial Infarction in Patients Presenting With Cerebrovascular Accident in a Tertiary Care Centre in Eastern India

**DOI:** 10.7759/cureus.c75

**Published:** 2022-09-29

**Authors:** Jaymala Mishra, Abhay Kumar, Santosh Kumar, Siddharth Singh, Santosh Kumar Nayan, Anand Dev

**Affiliations:** 1 Emergency Medicine, Indira Gandhi Institute of Medical Sciences, Patna, IND

This article has been corrected to add five additional authors who were not included prior to publication by the submitting author, Siddharth Singh. The submitting author greatly regrets this error.

